# Fowl adenovirus 8a isolated from chickens with runting and stunting syndrome induces inclusion body hepatitis and hepatitis-hydropericardium syndrome in chicken embryos

**DOI:** 10.14202/vetworld.2024.2556-2566

**Published:** 2024-11-13

**Authors:** Luis Nuñez, Silvana Santander-Parra, Marcia Catroxo, Claudete Serrano Astolfi-Ferreira, Anthony Loor-Giler, Antonio Piantino Ferreira

**Affiliations:** 1 Facultad de Ciencias de la Salud, Carrera de Medicina Veterinaria, Universidad de Las Américas (UDLA), Antigua Vía a Nayón S/N, Quito EC 170124, Ecuador; 2 One Health Research Group, Universidad de Las Américas (UDLA), Antigua Vía a Nayón S/N, Quito EC 170124, Ecuador; 3 Laboratory of Avian Diseases, School of Veterinary Medicine and Animal Science, Department of Pathology, University of São Paulo, São Paulo, Brazil; 4 Laboratory of Electron Microscopy, Center for Research and Development of Animal Health, Instituto Biologico, Av. Cons. Rodrigues Alves, 1252, 04014-002, São Paulo, SP, Brazil; 5 Laboratorios de Investigación, Dirección general de Investigación, Universidad de las Américas (UDLA), Antigua Vía a Nayón S/N, Quito EC 170124, Ecuador; 6 Facultad de Ingeniería y Ciencias Aplicadas, Carrera de Ingeniería en Biotecnología, Universidad de Las Américas (UDLA), Antigua Vía a Nayón S/N, Quito EC 170124, Ecuador

**Keywords:** chicken embryonated eggs, fowl adenovirus, hepatitis-hydropericardium syndrome, inclusion body hepatitis, runting and stunting syndrome

## Abstract

**Background and Aim:**

Fowl adenovirus (FAdV) is the etiological agent of inclusion body hepatitis (IBH) and hepatitis-hydropericardium syndrome (HHS) in poultry. It is also detected in chickens with runting and stunting syndrome (RSS). FAdV has been detected worldwide, and genotypes 8a, 8b, and 11 have been identified in chickens with enteric problems in Brazil. Nevertheless, none of them have been isolated; therefore, these viruses propagate; thus, the viral behavior and pathogenicity are unknown in Brazil. This study aimed to isolate FAdV from the enteric content of chickens affected by RSS.

**Materials and Methods:**

Enteric content samples from chickens affected with RSS and a positive polymerase chain reaction (PCR) test for FAdV were inoculated into specific pathogen-free chicken embryonated eggs (CEEs) through the yolk and chorioallantoic membrane routes on 8 and 10 days of age, respectively and the eggs were incubated for five days for viral isolation.

**Results:**

The embryos exhibited dwarfism, beak atrophy, and pale claws. In addition, some embryos displayed edema and gelatin-like characteristics. The liver exhibited hepatomegaly and multiple necrotic foci, resembling the appearance of nutmeg. In addition, the kidneys appeared enlarged and pale. After 8 days of incubation, the hearts of the inoculated embryos showed hydropericardium. Microscopic evaluation revealed the presence of hepatitis, which was characterized by the presence of intranuclear inclusion bodies and cellular necrosis. Viral FAdV particles were observed in kidney cells using electron microscopy. Viral DNA was detected in the embryos in all three passages, and viral gene copies were also measured in some organs, with high FAdV gene copies detected in the spleen and bursa beyond the liver.

**Conclusion:**

The molecular characterization of FAdV revealed that the isolated strain belonged to genotype 8a of FAdV. Here, FAdV-8a from chickens infected with RSSs produced IBH/HHS in CEE, and FAdV-8a detected in RSS outbreaks, in addition to producing IBH/HHS in chicken embryos, could be a possible viral agent that causes IBH/HHS in chickens.

## Introduction

Inclusion body hepatitis (IBH) and hepatitis-hydropericardium syndrome (HHS) are diseases that affect poultry worldwide [1–6]. The principal agent of this syndrome is fowl adenovirus (FAdV), and some virus serotypes have been detected in outbreaks of the disease [7, 8]. FAdV is a virus with a double-stranded DNA genome that is approximately 43–46 kb long and encodes 10 major structural proteins in the virion and non-structural proteins [7, 9–11]. FAdVs belong to the *Aviadenovirus* genus of *Adenoviridae* family. FAdV is divided into 5 species (FAdV-A to FadV-E) based on their restriction enzyme digestion pattern and also divided into 12 serotypes (FAdV-1 to 8a and 8b to 11) identified by cross-neutralization testing [7, 8, 12]. The principal serotypes related to IBH/HHS are 4, 7, 8a, 8b, and 11 and were detected and isolated in several countries around the world from cases of IBH [13–17]. This condition is characterized by accumulating clear to yellow fluid in the pericardial sac, nephritis, and hepatitis. The affected liver shows yellow to white areas and necrotic foci; microscopically, intranuclear bodies are observed beyond the necrotic cells, which characterize this disease [1, 18, 19]. Moreover, FAdV is associated with enteric problems that are detected in the intestinal contents of chickens with severe enteritis and those affected by runting and stunting syndrome (RSS) [20, 21]; nevertheless, experimental infections with FAdV were not conducted to determine whether the virus is responsible for the development of these enteric problems. FAdV is rapidly spread by the fecal and oral routes and through vertical transmission to progeny; thus, the presence of FAdV in fezzes is very important for virus spread [9]. At present, molecular techniques are the most commonly used assays to detect and characterize FAdV [7, 8, 22]; however, enzyme-linked immunosorbent assay [23–26] and electron microscopy (EM) are also used. Nonetheless, the gold standard method for FAdV diagnosis is viral isolation from chicken embryonated eggs (CEEs), in which the virus produces hemorrhagic, edematic, and stunted embryos using primarily the chorioallantoic membrane (CAM) [18, 27]. The liver is the most affected organ. During viral propagation, the liver exhibits necrotic foci and petechial hemorrhages, and viral particles of FAdV are visualized using electronic microscopy in the nuclei of hepatocytes, which are responsible for the formation of inclusion bodies [18, 28].

In South America, FAdV was detected in Chile [2], Peru [29], and Ecuador [30] and was associated with IBH and enteric disease. In Brazil, the virus was also detected in cases of IBH and RSS, which are associated with high mortality rates [20, 21, 31]. Previous reports of IBH in Brazil have shown lesions characterized by an enlarged liver with the presence of white areas of necrosis and straw-colored liquid in the pericardial sac [32]. In recent years, there has been a noticeable increase in cases of similar diseases in Brazilian poultry. Genotypes 8a, 8b, and 11 of FAdV have been identified and characterized through molecular detection [21]. The high identification of FAdV in the fezzes of Brazilian chicken flocks indicates the importance of investigating the behavior of the virus from the outset of isolation.

Therefore, this study aimed to isolate FAdV from enteric tissue, determine macroscopic and microscopic lesions in organs, and molecularly characterize the virus.

## Materials and Methods

### Ethical approval

All procedures conducted in the present research were performed in accordance with the guidelines and with the approval of the Committee for the Care and Use of Laboratory Animal Resources of the School of Veterinary Medicine, University of Sao Paulo, Brazil (number 2437/2011).

### Study period and location

The study was conducted from March 2020 to August 2023 using enteric content from chickens affected with RSS from Sao Paulo, Brazil. The samples were processed in the Laboratory of Avian Diseases, College of Veterinary Medicine, University of Sao Paulo.

### Samples

In the present study, a sample of polymerase chain reaction (PCR)-positive enteric content unique to FAdV was used, according to a study of enteric viruses in chickens published by our working group [32], in which all enteric content samples were tested for chicken astrovirus (CAstV), avian nephritis virus (ANV), avian rotavirus (ARoV), avian reovirus (AReV), infectious bronchitis virus (IBV), chicken parvovirus (ChPV), and FAdV. The sample was denominated USP 400-10A and subjected to inoculation in specific pathogen-free-CEEs (SPF-CEEs) for virus isolation, genotyping, and determination of pathological, macroscopic, and microscopic features caused by the virus in the embryos, which is consistent with the subsequent procedures.

### FAdV isolation from SPF-CEE

#### Inoculum

One milliliter of 0.1 M phosphate-buffered saline solution (PBS), pH 7.2, was added to a 2 mL microtube along with the same quantity of sample (enteric content), frozen at −80°C for 10 min, thawed for 1 min at 56°C, and subsequently homogenized. This procedure was repeated 3 times, after which the samples were centrifuged at 12,000× *g* for 30 min at 4°C. The supernatant was collected and filtered through a 0.22 μm pore size filter (Millipore™ by Merck, Darmstadt, Germany), and the inoculum was injected into the SPF-CEE.

#### Inoculation of FAdV in SPF-CEEs

The obtained inoculum (200 μL) was injected into each of the four SPF-CEEs after 7 days of incubation using the yolk sac route and in the same manner into four SPF-CEEs after 10 days of incubation using the CAM route. Eggs infected through each inoculation route were incubated at 37°C for 5 days and were checked daily for viability. The live and dead embryos were then necropsied, and the internal organs were partially harvested for nuclear acid extraction. The remaining organs were macerated and used for inoculum preparation for the next passages. In each passage, the inoculum from the previous passage was prepared from embryo tissues, and 200 μL were inoculated in the yolk sac or CAM in each SPF-CEE. The same number of SPF-CEEs and inoculation routes were used for the mock group, where 200 μL of 0.1 M PBS (pH 7.2) were injected into each egg.

#### Postmortem examination

As mentioned above, 5 days after inoculation, live and dead SPF-CEEs were slaughtered and subjected to a postmortem examination in which the presence or absence of macroscopic injuries was observed on the embryo body, supporting membranes and internal organs of the celomatic cavity. From each infected and non-infected SPF-CEE, the bursa, spleen, thymus, heart, proventriculus, jejunum, gizzard, liver, kidney, body embryo, and chorioallantoic membrane (MCA) were collected, and allantoic liquid was extracted.

### Sample processing

DNA extracted from samples of the three passages of SPF-CEEs inoculated by the yolk sac route and the samples from the two first passages of SPF-CEEs inoculated by the CAM route were tested to detect FAdV using conventional PCR. The samples from the third passage of SPF-CEEs inoculated by the CAM route were also analyzed for FAdV, and their viral load was determined in each organ collected using quantitative PCR (qPCR). DNA extracted from the inoculum was also used for FAdV genotyping. The embryonic organs were visualized to determine the presence of macroscopic alterations; the embryonic liver was examined microscopically (Nikon E200, Melville, USA) at 4× and 40× magnifications. for the presence of cell injuries, and the kidneys were evaluated ultra microscopically (Philips, Model EM 208, AMsterdam, Netherlands) at 25k and 50k magnification for the presence of viral particles inside the cells. The extracted DNA, embryos, and egg support membranes of the mock group were analyzed using the methods described here for the infected group.

### Histopathology

Liver tissue from the mock embryo group and embryos obtained from both inoculation routes were collected and fixed with 10% buffered formalin. Subsequently, the tissues were processed, embedded in paraffin, sectioned to a thickness of 5 μm, fixed, and stained with hematoxylin and eosin. All sections were analyzed using a light microscope (Nikon E200, Melville, USA) at 4× and 40× magnifications.

### Molecular analyses

A piece of approximately 50 mg of liver, jejunum, kidney, thymus, heart, proventriculus, gizzard, bursa, body embryo or CAM, or the whole spleen, 250 μL of collected amniotic liquid, or 250 μL of inoculum was placed in a 2 mL sterile microtube. The organs were disrupted using Tissue Lyser LT (Qiagen® - Straße 1, 40724 Hilden Germany) and immediately subjected to nucleic acid extraction.

### Nucleic acid extraction

DNA was extracted from 250 μL of inoculum, 250 μL of amniotic liquid, 50 mg of harvested organs, or the whole spleen using TRIzol reagent (Invitrogen by Life Technologies, Carlsbad, CA, USA) according to the manufacturer’s instructions.

### Molecular detection of FAdV

#### PCR for FAdV detection

In all passages of the virus in the SPF-CEEs of both routes of inoculation, except the third passage in the SPF-CEEs using the CAM route of inoculation, FAdV was tested in the inoculum (pooled organs) employing end-point PCR as previously described by Meulemans *et al*. [8], with some modifications in the annealing temperature. FAdV was also verified in the passages of SPF-CEEs in the mock group.

The DNA extracted (2 μL) from each organ or allantoic liquid was added to 23 μL of a mixture containing 0.5 μM of each primer (Table-1) [8, 22, 33–36], 2.5 μL of 10× buffer, 4 μL of 1.25 mM deoxynucleotide triphosphates (dNTPs), 37.5 mM MgCl_2_, and 1.0 U of Platinum Taq DNA polymerase (Invitrogen by Thermo Fisher Scientific, Carlsbad, CA, USA). The reaction was run under the same conditions described by Meulemans *et al*. [8]. A negative control was prepared by substituting DNA with 2 μL ddH_2_O, and a non-template control (NTCs) was used.

**Table-1:** Primer sequences used in this study.

Virus	Target gene	Primer name	Primer sequence	Amplicon size (bp)	Assay	References
ChPV	NS	PVA-F	5′-GCA ACT AAC CTG ACC GTG TG-3′	96	qPCR	[34]
		PFA-R	5′-CCC GGA TTC AGA ACC AGT AT-3′			
FAdV	52 K and pIIIa	52K-fw	5′-ATG GCK CAG ATG GCY AAG G-3′	176		[22]
		52K-rv	5′-AGC GCC TGG GTC AAA CCG A-3′			
		52K-F	5′-TGT ACG AYT TCG TSC ARA C-3′	773	PCR	
		52K-R	5′-TAR ATG GCG CCY TGC TC-3′			
	Hexon	Hexon A	5’-CAARTTCAGRCAGACGGT-3’	897		[8]
		Hexon B	5’-TAGTGATGMCGSGACATCAT-3’			
CAstV	ORF 1b	CASpol 1F	5’-GAYCARCGAATGCGRAGRTTG-3’	362		[33]
		CASpol 1R	5’-TCAGTGGAAGTGGGKARTCTAC-3’			
ANV	ORF 1b	ANVpol 1F	5’-GYTGGGCGCYTCYTTTGAYAC-3’	473		
		ANVpol 1R	5’-CRTTTGCCCKRTARTCTTTRT-3’			
ARoV	NSP4	NSP4-F30	5’-GTGCGGAAAGATGGAGAAC-3’	630		
		NSP4-R660	5’-GTTGGGGTACCAGGGATTAA-3’			
IBV	UTR	UTR 11	5’-ATGTCTATCGCCAGGGAAATGTC-3’	179		[35]
		UTR 44	5’-GGGCGTCCAAGTGCTGTACCC-3’			
		UTR 33	5’-GCTCTAACTCTATACTAGCCTA-3’			
AReV	S4	S4-F13	5’-GTGCGTGTTGGAGTTTCCCG-3’	1120		[36]
		S4-R1133	5’-TACGCCATCCTAGCTGGA-3’			

ChPV=Chicken parvovirus, FAdV=Fowl adenovirus, CAstV=Chicken astrovirus, ANV=Avian nephritis virus, ARoV=Avian rotavirus, IBV=Infectious bronchitis virus, qPCR=Quantitative polymerase chain reaction

#### qPCR for the detection and quantification of FAdV

FadV was examined in the last passage of the virus in SPF-CEEs inoculated through the CAM route, and the viral load distribution among the tissues was determined using the qPCR method described by Günes *et al*. [22], with some modifications in the annealing temperature. FAdV was also tested in the third passage of SPF-CEEs in the mock group using the method described here.

DNA was obtained from samples as described above and subjected to qPCR. The reactions were prepared using a mixture containing 10 μL of PowerUp™ SYBR^®^ green master mix (2×) (Applied Biosystems by Thermo Fisher Scientific, Carlsbad, CA, USA), 0.5 μM of each primer (Table-1), 1 μL of DNA from each sample and UltraPure™ DNase/RNase-free distilled dH_2_O (Invitrogen by Life Technologies) to a final volume of 20 μL. NTCs were included, and a negative control was prepared by substituting the DNA with dH_2_O. Reactions were cycled using a 7500 fast real-time PCR system (Applied Biosystems by Thermo Fisher Scientific) in standard mode with a hot start step at 95°C for 2 min, followed by 40 cycles at 95°C for 15 s, 60°C for 15 s, and 72°C for 1 min. A dissociation curve (melting) was obtained in three steps: 95°C for 15 s, followed by a decrease to 60°C for 1 min and a gradual increase in temperature (0.3°C) up to 95°C.

To determine the assay’s sensitivity, endpoint PCR was performed to amplify a portion of the 52 K and pIIIa genes of FAdV using a previously described method [22]. The obtained amplicon was purified using CleanSweep PCR Purification (Applied Biosystems by Thermo Fisher Scientific) according to the manufacturer’s instructions and quantified using a NanoDrop spectrophotometer (Thermo Fisher Scientific, Carlsbad, CA, USA). Using the web tool DNA copy Number and dilution Calculator (Thermo Fisher Scientific), the quantity of DNA necessary to prepare the first dilution with a known quantity of DNA copies was calculated. Then, 10-fold dilutions were prepared to determine the sensitivity and amplification efficiency of the qPCR assay.

### Molecular characterization of FAdV

DNA extracted from the inoculum of each passage was also subjected to endpoint PCR for FAdV detection using the method described by Meulemans *et al*. [8], with some modifications in the annealing temperature as described above. Therefore, a portion of the HEXON gene was amplified and purified using CleanSweep PCR Purification (Applied Biosystems by Thermo Fisher Scientific) according to the manufacturer’s instructions. Each purified amplicon was sequenced with forward and reverse primers using a BigDye^®^ terminator v3.1 cycle sequencing kit (Applied Biosystems by Thermo Fisher Scientific). The sequencing was performed using an ABI 3730 DNA analyzer (Applied Biosystems, Thermo Fisher Scientific). The obtained electropherograms were edited using the Geneious 2019.2.1 Software package (Biomatters Ltd., Auckland, New Zealand) and analyzed using Basic Local Alignment Search Tool (BLAST) (https://blast.ncbi.nlm.nih.gov/Blast.cgi) to determine their similarity with other sequences deposited in GenBank. The obtained consensus sequence was aligned with other sequences of FAdV using the ClustalX 2.2.1 software package (http://www.clustal.org/clustal2/), and the similarity of nucleotides (NT) and amino acids (AA) was inferred using the BioEdit Sequence Alignment Editor 7.2.5 software package (https://bioedit.software.informer.com/versions/). The phylogenetic tree was constructed using a neighbor-joining statistics method, together with a p-distance substitution model and a phylogeny test bootstrap model with 1000 replicates that were integrated into the MEGA version 7 software package (https://www.megasoftware.net/) [37].

### Transmission electron microscopy (TEM)

A piece of approximately 1 mm of kidney tissue from an embryo infected with FAdV through the CAM route at the third passage was fixed with 2.5% glutaraldehyde in 0.1 M PBS (pH 7.2) and subsequently processed for resin inclusion and positive staining with uranyl acetate and lead citrate. Samples were observed under an electron microscope (Philips, Model EM 208, Amsterdam, Netherlands) to assess the presence of viral particles.

### GenBank accession number

The accession number of part of the Hexon gene of FAdV-8a was deposited in GenBank under the accession number USP 400-10 A (MN453821.1).

### Differential diagnosis

The molecular diagnostics of other enteric viruses were applied to all passages in the mock and SPF-CEE-infected groups. The tested viruses were CAstV, ANV, and ARoV according to the method described by Day *et al*. [33], ChPV using the protocol described by Nuñez *et al*. [34], IBV using the method described by Cavanagh *et al*. [35], and AReV according to the protocol described by Pantin-Jackwood *et al*. [36] (Table-1).

## Results

### Isolation of FAdV from SPF-CEEs

The USP 400-10A inoculated sample was propagated in and isolated from SPF-CEEs using both inoculation routes. The confirmation of FAdV isolation was based on the visualization of viral particles in kidney tissues, macroscopic and microscopic injuries in the body and organs, and virus detection in all samples harvested at each passage of both inoculation routes using conventional and qPCR. FAdV was isolated from SPF-CEEs generated using the yolk sac or CAM route.

### Postmortem examination

**Figure-1:**
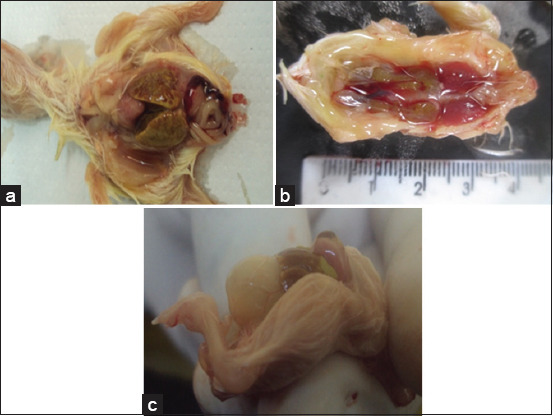
Macroscopic injuries in SPF-CEEs infected with FAdV-8a. (a) Nutmeg liver from an SPF-CEE inoculated with FAdV-8a via the CAM route after 15 days of incubation (third passage). (b) Enlarged and pale kidneys from an SPF-CEE inoculated by the yolk sac route after 13 days of incubation (third passage). (c) SPF chicken embryo infected with FAdV-I using the yolk sac route showing hepatitis (yellow foci) and hydropericardium at 13 days of incubation (third passage). SPF-CEEs=Specific pathogen-free chicken embryonated eggs, FAdV=Fowl adenovirus, CAM=Chorioallantoic membrane.

In the postmortem examination, the embryos of each passage generated from both inoculation routes showed dwarfism, beak atrophy, pale claws, and thin skin, which facilitated observation of the internal organs, and some embryos also showed edema and gelatin. The liver presented with hepatomegaly and multiple necrotic foci resembling nutmeg. The intestines were filled with liquid, and the kidneys were enlarged and pale. The hearts of the inoculated embryos presented as hydropericardium 8 days after inoculation (Figure-1 and Table-2). The other organs did not exhibit any macroscopic alterations. The embryos of the mock group did not exhibit macroscopic alterations.

**Table-2:** Pathological and histopathological findings of SPF-CEEs infected with FAdV-8a.

Passage in SPF-CEEs	Inoculation route	Macroscopic lesions				Microscopic lesions		
	
		Nutmeg liver	Intestines filled with liquid	Hydroperi-cardium	Enlarged and pale kidneys	Necrotic hepatitis	IBH	Heterophile/Lymphocyte infiltrate
First	YS	+ (3/4)	+ (4/4)	+ (1/4)	+ (2/4)	NP	NP	NP
	CAM	+ (3/4)	+ (4/4)	+ (0/4)	+ (1/4)	NP	NP	NP
Second	YS	+ (4/4)	+ (4/4)	+ (3/4)	+ (4/4)	NP	NP	NP
	CAM	+ (4/4)	+ (4/4)	+ (4/4)	+ (4/4)	NP	NP	NP
Third	YS	+ (4/4)	+ (4/4)	+ (4/4)	+ (4/4)	+ (4/4)	+ (4/4)	+ (4/4)
	CAM	+ (4/4)	+ (4/4)	+ (4/4)	+ (4/4)	+ (4/4)	+ (4/4)	+ (4/4)

IBH=Inclusion body hepatitis, NP=Not performed, +=positive, SPF-CEEs=Specific pathogen-free chicken embryonated eggs, YS=yolk sac, CAM=Chorioallantoic membrane, FAdV=Fowl adenovirus

### Histopathology

**Figure-2:**
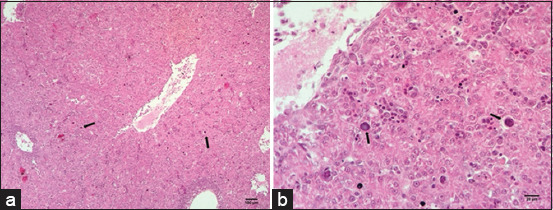
Microscopic lesions in the nutmeg liver of an SPF-CEE. (a and b) Hepatitis shows necrotic cells, apoptosis, bile duct proliferation, and the presence of intranuclear inclusion bodies (Arrows). SPF-CEE=Specific pathogen-free chicken embryonated egg.

Liver sections from embryos generated using both inoculation routes showed microscopic alterations characterized by bile duct proliferation, hepatic cell necrosis, apoptosis, cells with shape degeneration, eosinophilic intranuclear inclusion bodies, and the presence of heterophiles and plasma cell infiltration (Figure-2). Liver fragments from the mock group did not show any microscopic alterations.

### Detection of FAdV in SPF-CEEs

FAdV was detected using conventional PCR in the inoculum of each passage from both inoculation routes, except in the third passage of SPF-CEEs inoculated by the CAM route, where conventional PCR was not performed (Table-3). FAdV was not detected in the mock group.

**Table-3:** Distribution of FAdV-8a in the tissues and support membranes of SPF-CEEs

Tissues	Passage 1		Passage 2		Passage 3		

	PCR-FAdV-I		qPCR-FAdV		VC/mg		
		
	YSr	CAMr	YSr	CAMr	YSr	CAMr	VC
Pooled organs	+	+	+	+	+	NP	-
Allantoic liquid	NP	NP	NP	NP	NP	+	21.6
CAM	NP	NP	NP	NP	NP	+	27
Bursa	NP	NP	NP	NP	NP	+	73949.4
Spleen	NP	NP	NP	NP	NP	+	74611.6
Thymus	NP	NP	NP	NP	NP	+	1060.2
Heart	NP	NP	NP	NP	NP	+	366.1
Embryonic body	NP	NP	NP	NP	NP	+	6932.7
Proventriculus	NP	NP	NP	NP	NP	+	2848.5
Duodenum	NP	NP	NP	NP	NP	+	2569.3
Gizzard	NP	NP	NP	NP	NP	+	9790.2
Liver	NP	NP	NP	NP	NP	+	3507.8
Kidney	NP	NP	NP	NP	NP	+	1047.2

SPF-CEE=Specific pathogen-free chicken embryonated eggs, YSr=Yolk sac route, CAMr=Chorioallantoic membrane route, CAM=Chorioallantoic membrane, VC=Viral copies, mg=Milligram, NP=Not performed, +=Positive, FAdV=Fowl adenovirus, qPCR=Quantitative polymerase chain reaction

### The detection and quantification of FAdV in SPF-CEEs

A previously published qPCR assay [22] was used to detect and quantify FAdV, and the assay detected and quantified a load of 10–10^8^ copies of DNA. The limit of detection and the limit of quantification were 10 DNA copies. The melting curve showed a unique peak at 85.57°C, and primer dimers were not observed. The standard curve has an efficiency of 96.1%.

In the third passage of FAdV in SPF-CEEs inoculated via the CAM route, the viral load was determined among the organs, and a high viral load was detected in all organs, principally in the bursa and spleen, followed by the gizzard, liver, proventriculus, duodenum, and thymus. CAM and allantoic liquids had the lowest viral load (Table-3). Based on these results, FAdV replicated in many organs; however, the highest viral load was observed in lymphopoietic organs. FAdV was not detected in any passage of SPE-CEEs generated using either route of inoculation.

### Molecular characterization of FAdV isolated from SPF-CEEs

**Figure-3:**
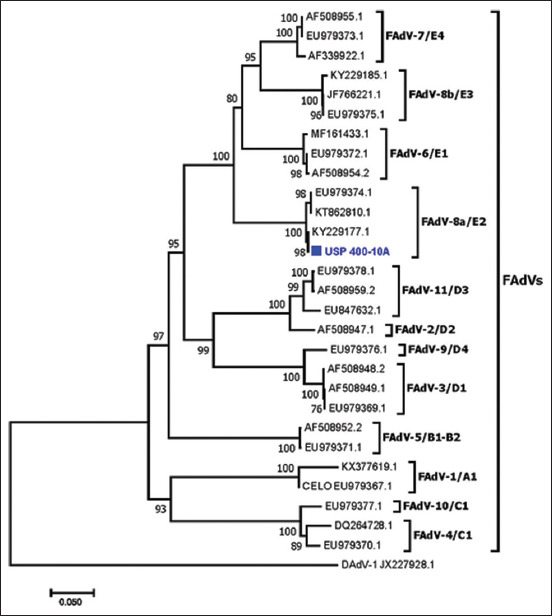
A phylogenetic comparison was performed using the sequence of the HEXON gene obtained in the present study and other sequences of FAdV for many serotypes present in GenBank. Sequences were aligned using the CLUSTAL W method in ClustalX2 2.1. The phylogenetic tree was constructed using the MEGA 7 software package. Numbers along the branches are bootstrap values for 1000 replicates. The scale bar represents the number of substitutions per site. Duck adenovirus I was used as the outgroup. The blue sequence marked with a cube was obtained in this study from FAdV isolated from SPF-CEEs. SPF-CEEs=Specific pathogen-free chicken embryonated eggs, FAdV=Fowl adenovirus.

The electropherograms generated here assembled a sequence of 903 bp, and the BLAST tool revealed high similarity with other FAdV sequences deposited in GenBank. The isolated USP 400-10A shares high similarity of NT (99.1%–100%) and AA (99.4%–100%) with sequences of genotype 8a from FAdV and lower similarity of NT (80%–81%) and AA (84.3%–85.3%) with genotype 8b; similar results were obtained for the sequences from the other FAdV genotypes (Table-4). The phylogenetic analysis grouped the sequence obtained here with the FAdV-8a group with a bootstrap value of 100%, and the other genotypes were also clustered in their corresponding group (Figure-3). The molecular analyses determined that the sample isolated here belonged genetically to FAdV genotype 8a.

**Table-4:** Comparison of the nucleotide and amino acid identities of the Brazilian FAdV-8a sequence with those of other FAdVs.

No.	Genotype	Sequences	Amino acid content (%)														

			FAdV-1	FAdV-2	FAdV-3			FAdV-4		FAdV-5		FAdV-6			FAdV-7		
						
			1	2	3	4	5	6	7	8	9	10	11	12	13	14	15
1	FAdV-1	EU979367.1	-	57.7	60.8	60.8	60.8	61.9	62.4	62	62.5	58.7	58.7	58.2	59.7	59.7	60.8
2	FAdV-2	AF508947.1	60	-	78.6	78.6	78.6	52.2	53.2	67.5	68	78.7	78.7	78.2	77.2	77.2	77.7
3	FAdV-3	AF508949.1	61.9	72.5	-	100	100	53.7	54.2	68	68.5	79.2	79.2	78.7	77.7	77.7	79.2
4		EU979369.1	61.9	72.5	100	-	100	53.7	54.2	68	68.5	79.2	79.2	78.7	77.7	77.7	79.2
5		AF508948.2	62	72.6	99.8	99.8	-	53.7	54.2	68	68.5	79.2	79.2	78.7	77.7	77.7	79.2
6	FAdV-4	DQ264728.1	63.6	55.6	55	55	54.8	-	94.7	54	54.5	53.2	53.2	52.7	53.2	53.2	53.7
7		EU979370.1	65.4	56.5	56.3	56.3	56.1	95.8	-	55	55.5	52.2	52.2	51.7	53.2	53.2	54.2
8	FAdV-5	AF508952.2	63.2	65.6	63.9	63.9	64.1	57.7	59.2	-	99.5	68.5	68.5	68	67.5	67.5	68.5
9		EU979371.1	63.2	65.7	63.9	63.9	64.1	57.9	59.4	99.8	-	69	69	68.5	68	68	69
10	FAdV-6	MF161433.1	63.1	70.3	69.1	69.1	69.2	57.4	58.4	66.9	67.1	-	100	99.4	91.4	91.4	90.9
11		EU979372.1	62.9	70.3	69.1	69.1	69.2	57.7	58.7	67.1	67.3	99.3	-	99.4	91.4	91.4	90.9
12		AF508954.2	62.8	70.1	68.9	68.9	69.1	57.5	58.5	66.9	67.1	99.1	99.8	-	90.9	90.9	90.4
13	FAdV-7	AF508955.1	63.6	68.7	66.7	66.7	66.9	57.9	58.9	66.6	66.5	86	85.9	85.7	-	100	96.9
14		EU979373.1	63.6	68.7	66.7	66.7	66.9	57.9	58.9	66.6	66.5	86	85.9	85.7	100	-	96.9
15		AF339922.1	64.1	68.9	66.9	66.9	67.1	58	59.4	66.5	66.6	86.4	86.2	86	98.1	98.1	-
16	FAdV-8a	USP 400-10 A	61.7	70.1	67.7	67.7	67.9	57.1	58.5	66.9	66.7	82.5	82.7	82.5	82.5	82.5	82.5
17		KY229177.1	61.7	70.1	67.7	67.7	67.9	57.1	58.5	66.9	66.7	82.5	82.7	82.5	82.5	82.5	82.5
18		EU979374.1	61.5	70.3	67.1	67.1	67.2	57.3	58.5	66.7	66.6	81.9	82	81.9	81.9	81.9	81.9
19		KT862810.1	61.5	70.3	67.1	67.1	67.2	57.3	58.5	66.7	66.6	81.9	82	81.9	81.9	81.9	81.9
20	FAdV-8b	KY229185.1	60.6	68.1	67.2	67.2	67.4	55	56.2	62.8	62.6	80.7	80.7	80.5	87.5	87.5	87.5
21		JF766221.1	60.9	67.7	67.4	67.4	67.6	55.2	56.4	63.3	63.1	81.2	81.2	81	88	88	88
22		EU979375.1	60.9	67.7	67.4	67.4	67.6	55.2	56.4	63.3	63.1	81.2	81.2	81	88	88	88
23	FAdV-9	EU979376.1	62	73.8	94.6	94.6	94.4	55.5	56.5	63.4	63.4	68.6	68.6	68.4	67.1	67.1	66.9
24	FAdV-10	EU979377.1	65.3	56.3	55.8	55.8	55.6	93.9	95.5	58.9	59	58.5	58.7	58.5	58.7	58.7	59
25	FAdV-11	EU979378.1	61	94	75.5	75.5	75.7	55.5	56.5	64.2	64.4	70.6	70.6	70.4	68.1	68.1	68.7
26		AF508959.2	61.2	93.9	75.7	75.7	75.8	55.3	56.3	64.2	64.2	70.4	70.4	70.3	68.1	68.1	68.6
			% nucleotides														

**No.**	**Genotype**	**Sequences**	**Amino acid content (%)**														

			**FAdV-8a**				**FAdV-8b**			**FAdV-9**	**FAdV-10**	**FAdV-11**					
			
			**16**	**17**	**18**	**19**	**20**	**21**	**22**	**23**	**24**	**25**	**26**				

1	FAdV-1	EU979367.1	58.7	58.7	58.7	58.7	58.2	58.7	58.7	60.8	63.4	58.2	58.2				
2	FAdV-2	AF508947.1	78.7	78.7	79.2	79.2	76.2	75.7	75.7	79.1	53.2	92.3	92.3				
3	FAdV-3	AF508949.1	78.7	78.7	78.7	78.7	76.7	76.7	76.7	91.8	54.7	80.7	80.7				
4		EU979369.1	78.7	78.7	78.7	78.7	76.7	76.7	76.7	91.8	54.7	80.7	80.7				
5		AF508948.2	78.7	78.7	78.7	78.7	76.7	76.7	76.7	91.8	54.7	80.7	80.7				
6	FAdV-4	DQ264728.1	54.7	54.7	54.7	54.7	52.7	53.2	53.2	53.7	92.1	52.7	52.7				
7		EU979370.1	56.2	56.2	55.7	55.7	52.7	53.2	53.2	53.7	93.7	53.7	53.7				
8	FAdV-5	AF508952.2	68	68	68	68	67	67	67	67.5	55.5	67	67				
9		EU979371.1	68.5	68.5	68.5	68.5	67.5	67.5	67.5	68	56	67.5	67.5				
10	FAdV-6	MF161433.1	86.8	86.8	86.8	86.8	84.8	85.3	85.3	78.7	53.2	78.2	78.2				
11		EU979372.1	86.8	86.8	86.8	86.8	84.8	85.3	85.3	78.7	53.2	78.2	78.2				
12		AF508954.2	86.3	86.3	86.3	86.3	84.3	84.8	84.8	78.2	52.7	77.7	77.7				
13	FAdV-7	AF508955.1	85.3	85.3	84.8	84.8	89.3	89.8	89.8	76.7	52.2	77.2	77.2				
14		EU979373.1	85.3	85.3	84.8	84.8	89.3	89.8	89.8	76.7	52.2	77.2	77.2				
15		AF339922.1	85.8	85.8	85.3	85.3	90.4	90.9	90.9	77.2	52.2	78.2	78.2				
16	FAdV-8a	USP 400-10 A	-	100	99.4	99.4	84.8	85.3	85.3	77.2	56.2	78.7	78.7				
17		KY229177.1	100	-	99.4	99.4	84.8	85.3	85.3	77.2	56.2	78.7	78.7				
18		EU979374.1	99.1	99.1	-	100	84.3	84.8	84.8	77.2	56.2	79.2	79.2				
19		KT862810.1	99.1	99.1	100	-	84.3	84.8	84.8	77.2	56.2	79.2	79.2				
20	FAdV-8b	KY229185.1	80.5	80.5	80	80	-	99.4	99.4	76.2	52.7	76.2	76.2				
21		JF766221.1	81	81	80.5	80.5	99.4	-	100	76.2	53.2	75.7	75.7				
22		EU979375.1	81	81	80.5	80.5	99.4	100	-	76.2	53.2	75.7	75.7				
23	FAdV-9	EU979376.1	66.9	66.9	66.6	66.6	66.9	67.1	67.1	-	55.2	79.6	79.6				
24	FAdV-10	EU979377.1	58.5	58.5	58.6	58.6	56	56.2	56.2	56.3	-	53.2	53.2				
25	FAdV-11	EU979378.1	70.1	70.1	70.3	70.3	67.6	67.2	67.2	76.2	56.1	-	100				
26		AF508959.2	70.1	70.1	70.3	70.3	67.6	67.2	67.2	76.3	56	99.8	-				
			% nucleotides														

FAdV=Fowl adenovirus

### TEM

**Figure-4:**
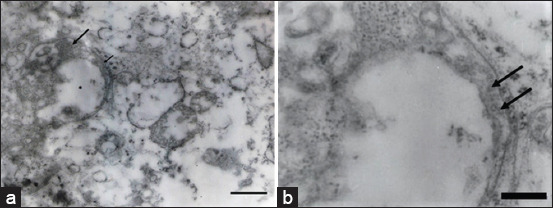
Transmission of electron micrographs of viral particles in the kidney infected with FAdV-8a through the yolk sac route after 13 days of incubation. (a) An empty nucleus (asterisk), with dilation of the outer membrane where viral particles adhere (arrows). Bar = 860 nm. (b) Higher-magnification of the image shown in (a). Viral particles 50 nm in diameter were located in the space formed by the dilation of the outer nuclear membrane (arrows). Bar: 110 nm. FAdV=Fowl adenovirus.

Part of the kidneys of embryos infected with FAdV-8a was subjected to TEM, and the kidney cells showed the presence of structures compatible with viral particles, confirming the presence of the virus in the tissue (Figure-4).

### Differential diagnosis

The samples of SPF-CEEs infected with FAdV-8a and those in the mock group were negative for ANV, CAstV, IBV, ARoV, ARe, and ChPV.

## Discussion

This study describes the isolation of FAdV-8a from chickens affected with enteric disease. The main pathological features of the virus in SPF-CEE are classical lesions in the liver (necrosis) with intranuclear inclusion bodies and hydropericardium in chick embryos during virus isolation.

Avian adenoviruses are divided into three general serotypes: *Aviadenovirus*, *Siadenovirus*, and *Atadenovirus*, while the *Aviadenovirus* species that affect birds are divided into 5 species (A–E) and 12 genotypes. Additional species have also been detected in turkeys, ducks, geese, and wild birds. Most *Aviadenovirus* species are subclinical and cause disease only when concurrent diseases are present, with the exception of some strains of FAdV-1 that cause erosion in the gizzard, FAdV-4 that causes HHS, and some species of FAdV species D (FAdV-2, FAdV-11) and E (FAdV-6) that cause IBH disease [9, 11, 38–42].

Adenoviruses are distributed around the world, including Brazil, where certain genotypes have been detected, including 8a, 8b, and 11 [21]; FAdV-8a is widely distributed in the United States [12], Australia [12], Malaysia [12], New Zealand [12], Canada [12], France [12], Mexico [12], Italy [12], Belgium [12], Hungary [12], South Korea [43], China [44, 45], Poland [46], and Spain [46]. In our study, research has revealed the presence of FAdV-8a in Brazil, as molecular studies indicate a high percentage of NT and AA similarity between the virus isolated here with other sequences described in the literature (Figure-3). As shown in the phylogenetic tree, the isolated USP 400-10A strain was grouped with other sequences of FAdV-8a with a bootstrap value of 100% and was the same as other genotypes that were clustered with the respective genotype, indicating that the virus isolated here belongs to genotype 8.

According to Radwan *et al*. [47], FAdV-8a is the primary agent causing IBH, and it is responsible for substantial economic losses because it also produces gizzard erosions [48, 49]. Nevertheless, strains were identified in Spain and China that were related to liver and hydropericardial lesions in both broilers and layers, causing mortality ranging from 5% to 20% under field conditions [17, 50].

Various methods have been developed for FAdV diagnosis, with PCR being the most frequently used method. Other methods include viral isolation, serotyping by virus neutralization, immunohistochemistry, and *in situ* hybridization [46]. Here, FAdV-8a was detected using PCR, qPCR, and TEM and propagated into CEEs.

The first report of FAdV in Brazil was related to chickens showing hepatitis and hydropericardium in Sao Paulo State [32], where most chicken farms are located. The aforementioned study did not determine the genotype of the adenovirus responsible for chicken disease; however, FAdV DNA was detected in some organs of the affected animals and, principally, in the liver [32]. Afterward, a molecular survey detected genotypes 8a, 8b, and 11 in chickens with enteric problems, principally with RSS [21, 51]; nevertheless, to date, in Brazil, FAdV has not been shown experimentally to be responsible for HHS and IBH. In the present study, we isolated FAdV-8a from the enteric content of chickens affected with enteric diseases, which causes hepatic lesions and hydropericardium in embryos from SPF-CEE; the origin of the FAdV strain suggests that fezzes disseminate FAdV-8a and might cause outbreaks of IBH and HHS in Brazilian poultry (data not published), resulting in substantial economic losses.

FAdV is mainly isolated and propagated using CEEs, where some routes of inoculation have been studied, with inoculation through MCA being the more sensitive route than the allantoic cavity [18, 52]. The yolk sac has also been used as an inoculation route for adenoviruses [53, 54]. Our sample was inoculated into embryonated eggs using the MCA route and yolk sac, and we detected macroscopic lesions as described in a previous study by Alemnesh *et al*. [18], such as liver, intestinal, kidney, and cardiac alterations. All these alterations were attributed to FAdV-8a, since viral particles were also identified in the embryos’ kidneys through TEM (Figure-4). In addition, microscopic necrosis of liver cells, apoptosis, and proliferation of bile ducts (Figure-2) were observed, which are consistent with the lesions reported by Alemnesh *et al*. [18] on chicken embryos inoculated using similar routes as described above in other studies. Chicken embryos also presented hydropericardium (Figure-1), a lesion typically found in chickens infected with FAdV. To the best of our knowledge, this report is the first to document this lesion in chicken embryos caused by FAdV-8a in Brazil. The virus causes severe damage to the liver that might decrease albumin levels in the blood, altering the pressures and triggering hydropericardium. Similar lesions were observed in SPF-CEEs inoculated with FAdV-9 through the CAM route, where the virus replicates very well in the CAM and produces intranuclear inclusion bodies in the cells [18]. The virus replicated in various organs in addition to the liver. These organs include the CAM, bursa of Fabricius, spleen, heart, fibroblast, proventriculus, duodenum, gizzard, and kidneys. Notably, all of these organs exhibited high viral copy numbers. However, the bursa and spleen had more viral gene copies, followed by the thymus, denoting that the virus affected lymphopoietic organs and altered innate immunity. A potential explanation for these findings is that chicken embryos naturally infected with FAdV through the vertical route hatch with immunosuppression and are susceptible to infection with other microbiological agents, leading to IBH/HHS and high economic losses through morbidity and mortality. Experimental infection in broiler chickens with FAdV-11 produces IBH, and the virus is distributed in organs other than the liver, such as the lungs, thymus, spleen, and kidneys [55]. A study by Grgić *et al*. [56] in experimentally infected chickens showed copies of the FAdV-4 or FAdV-8b genes in cecal tonsils, followed by the liver and bursa of Fabricius. All other organs exhibited the presence of viral DNA, and the virus-induced messenger RNA expression of the cytokines interleukin (IL)-10 and interferon-gamma (IFN-γ) in the liver, indicating that hepatocytes fight against the pathogen and decreased expression of IFN-γ and IL-18 in the spleen, resulting in reduced immunity in these organs [56–59]. Nevertheless, these changes in cytokine expression occurred in organs without any lesions because the FAdV-4 strain used did not cause pathological lesions; on the other hand, in the present investigation, the FAdV-8a strain caused large lesions in hepatic cells, and thus, more studies are needed to determine the effect of the isolated virus (USP 400-10A) on cytokine expression in experimentally infected chickens and in chicken embryos.

The isolation of several viruses requires three or more passages in the CEE. Nevertheless, in our study, we detected viral DNA using PCR [8, 21] from the first passage, indicating that the virus had a high adaptation to chicken embryo cells, providing an alternative for virus attenuation to develop a vaccine that might be used to protect against adenovirus infection in Brazilian chicken flocks and principally to avoid IBH/HHS.

## Conclusion

This study indicated that the FAdV under investigation belongs to genotype 8a. This strain, isolated from chickens with RSS, was found to cause IBH and HHS in chicken embryos during isolation. Furthermore, the study revealed that multiple organs, in addition to the liver, had a high viral copy number, with lymphopoietic organs displaying the highest viral load. These findings show that FAdV-8a is responsible for inducing IBH/HHS in chicken embryos and may lead to outbreaks of IBH/HHS in chickens.

## Authors’ Contributions

LN: Sample collection, conducted the study, and wrote the original manuscript. SSP: Participated in the execution, data analysis, and revision of the manuscript. MC: Participated in the execution of the study and revision of the manuscript. CSAF: Sample collection and revised the manuscript. ALG: Data analysis and revised the manuscript. APF: Project design, planning, and revised the manuscript. All authors have read and approved the final manuscript.
